# Incorporation of an intercostal catheter into a multimodal analgesic strategy for uniportal video-assisted thoracoscopic surgery: a feasibility study

**DOI:** 10.1186/s13019-021-01590-z

**Published:** 2021-07-31

**Authors:** Jian Wei Tan, Jameelah Sheik Mohamed, John Kit Chung Tam

**Affiliations:** 1 Department of Cardiac, Thoracic and Vascular Surgery, National University Heart Centre, National University Health Systems, Singapore, Singapore; 2 Department of Surgery, Yong Loo Lin School of Medicine, National University Singapore, 1E Kent Ridge Road, NUHS Tower Block, Singapore, 119228 Singapore

**Keywords:** Uniportal video-assisted thoracoscopic surgery (UVATS), Postoperative recovery after thoracic surgery, Subpleural analgesia, Intercostal catheter, ON-Q pain relief system

## Abstract

**Background:**

Well-controlled postoperative pain is essential for early recovery after uniportal video-assisted thoracoscopic surgery (UVATS). Conventional analgesia like opioids and thoracic epidural anaesthesia have been associated with hypotension and urinary retention. Intercostal catheters are a regional analgesic alternative that can be inserted during UVATS to avoid these adverse effects. This feasibility study aims to evaluate the postoperative pain scores and analgesic requirements with incorporation of an intercostal catheter into a multimodal analgesic strategy for UVATS.

**Methods:**

In this observational study, 26 consecutive patients who underwent UVATS were administered a multilevel intercostal block and oral paracetamol. All of these patients received 0.2% ropivacaine continuously at 4 ml/h via an intercostal catheter at the level of the incision. Rescue analgesia including etoricoxib, gabapentin and opioids were prescribed using a pain ladder approach. Postoperative pain scores and analgesic usage were assessed. The secondary outcomes were postoperative complications, days to ambulation and length of stay.

**Results:**

No technical difficulties were encountered during placement of the intercostal catheter. There was only one case of peri-catheter leakage. Mean pain score was 0.31 (range 0–2) on post-operative day 1 and was 0.00 by post-operative day 5. 16 patients (61.6%) required only oral rescue analgesia. The number of patients who required rescue non-opioids only increased from 1 in the first 7 months to 8 in the next 7 months. There were no cases of hypotension or urinary retention. Median time to ambulation was 1 day (range 1–2). Mean post-operative length of stay was 4.17 ± 2.50 days.

**Conclusions:**

Incorporation of an intercostal catheter into a multimodal analgesia strategy for UVATS is feasible and may provide adequate pain control with decreased opioid usage.

## Background

Pain management in thoracic surgery is essential to promote early recovery and avoid complications like urinary retention, atelectasis and pneumonia. For pain control after thoracotomy, thoracic epidural analgesia (TEA) and paravertebral blocks (PVB) have been regarded as the gold standards [1]. In recent years, owing to the success of minimally invasive thoracic surgery, there has been an increasing adoption of surgical techniques like uniportal video-assisted thoracoscopic surgery (UVATS). However, the optimal postoperative analgesia after UVATS is still undetermined.

Studies have demonstrated that both TEA and PVB provide adequate analgesic effect after major thoracic surgery, but the former may be associated with adverse effects such as urinary retention and hypotension [2–5]. Failure rates of TEA placement have also been reported to be up to 30% [6, 7]. In recent years, the placement of an intercostal catheter (ICC) has been explored as a regional analgesic option. The multihole catheter can be inserted at the uniport incision in an atraumatic manner under direct thoracoscopic vision. When connected to a single-use elastomeric pump, the catheter can deliver an anaesthetic continuously and independently. The pump is available in a variety of volumes. Depending on the model, the flow rate of the anaesthetic can be fixed or titrated with an attached controller. The ICC has been shown to achieve good postoperative pain control in abdominal and orthopaedic surgeries [8–10], but evidence on its utility in thoracic surgery is mixed [11–13].

There has been a shift away from systemic opioids to regional options for pain management after thoracic surgery [14, 15]. This is especially relevant in guidelines promoting the use of opioid-sparing multimodal analgesia from evidence-based perioperative care protocols such as the Enhanced Recovery After Surgery (ERAS®) pathway [16]. The objective of this feasibility study was to evaluate our hypothesis that utilizing an ICC as part of a multimodal analgesic strategy would provide adequate pain relief after UVATS, while reducing the usage of opioids.

## Materials and methods

### Study population

This case series was conducted at the National University Hospital, Singapore. 26 consecutive patients who underwent UVATS and received the ICC over a period of 14 months were included. The exclusion criteria were open thoracotomy, UVATS converted to open thoracotomy, reoperation and age below 21 years old.

### Operative procedure

UVATS was performed in all patients. A single 3–4 cm incision was made at an appropriate intercostal space along the anterior axillary line. The exact level of the incision was tailored and individualized to each patient, depended on various factors like the extent of resection, patient’s body habitus and width of intercostal space. Generally an incision at the 4th or 5th intercostal space was made for abnormalities in the upper lobe, which the 5th or 6th intercostal space was used for the lower lobe. As our standard practice, a total muscle-sparing technique was employed, where the latissimus dorsi, serratus anterior and intercostal muscles were preserved. Subperiosteal entry into the pleural space was obtained without any spreading, resection or fracture of ribs. We retracted the incision with a small plastic wound protector (Alexis wound protector/retractor, Applied Medical). A lubricating gel was applied on the wound protector to facilitate removal of any resected specimens. At the end of surgery, a chest tube was inserted via the same incision and attached to a drainage system. An indwelling urinary catheter, if inserted to monitor intraoperative fluid status, was removed at the end of the surgery.

### Analgesic strategy

All patients received a standardised analgesic regimen consisting of a single-shot intercostal block and paracetamol 1 g 6 hourly, starting on the day of surgery. Before wound closure, a bolus of 1 mg/kg of 0.5% bupivacaine was injected into the intercostal space at the level of the incision, two levels above and below, as well as along the incision. This multilevel block was performed to fully cover overlapping innervation from the intercostal nerves, and to ensure adequate analgesia in the immediate postoperative period before the ICC takes into full effect. Rescue analgesics such as etoricoxib 90 mg once daily, gabapentin 300 mg in the evening, tramadol 50 mg 3 times a day, morphine 5 mg once and oxycodone 5 mg once, were prescribed by the thoracic surgery team when required. Intravenous patient-controlled analgesia (PCA) with a demand dose of morphine 1 mg was selectively prescribed and ceased by the acute pain team depending on their daily assessment of the patient.

### Placement and management of intercostal catheter

The administration of the ICC (ON-Q Pain Relief System, Halyard Health) was standardised across all patients. The ICC was inserted at the level of the same intercostal space as the incision. The provided blunt tunneler was inserted via the incision along the inferior rib border and used to dissect the extrapleural space under direct thoracoscopic vision, while keeping the pleura intact. The ON-Q multihole catheter was then introduced over the tunneler, followed by the removal of the tunneler. Ropivacaine was then administered via the catheter to visualise an intercostal bulge between the ribs. Subsequently, the catheter was connected to a 400 ml elastomeric pump filled with 0.2% ropivacaine solution as per the manufacturer’s recommendation, at a fixed flow rate of 4 ml/h. The pump was either kept for 4 days until it was depleted, or removed earlier if the patient was fit for discharge earlier. The ICC tubing was secured to the skin by a 3 M™ Tegaderm™ Transparent Film Dressing and the pump was placed in a small portable pouch that patients can carry around. All patients were regularly reviewed by our specialised thoracic nurse for symptoms of ropivacaine toxicity and the ICC tubing would be clamped in the event of an emergency. Our thoracic nurse also checked for the patency of the ICC and troubleshooted as appropriate, such as unkinking the tubing and reapplying another Tegaderm™ to secure it to the skin.

### Data collection

We performed a retrospective review of electronic medical records to obtain baseline patient characteristics, incidence of postoperative complications, duration of chest tube drainage, time to ambulation and length of stay. All patients were admitted to the cardiothoracic high dependency unit (HDU) after surgery, and were transferred to the general ward from the second postoperative day (POD) onwards when deemed suitable by the thoracic surgery team. Starting from POD 1, all patients were also assessed by a physiotherapist daily on their ability to ambulate. A pain score using a visual analogue scale (VAS) with numeric ratings of 0–10 was used by trained nurses to assess the level of pain, and this was recorded electronically. On POD 1, pain scores were recorded every 4 h in the HDU and the mean was calculated. For subsequent POD, the mean of all pain scores reported on that day was obtained. The type and amount of rescue analgesics required was also recorded electronically in a standardised fashion.

### Statistical analysis

All collected data was analysed with Statistical Package for the Social Sciences Version 22 (SPSS Inc., Chicago IL, USA). For categorical variables, the frequency and percentage are reported while the mean ± standard deviation or mean and range were calculated for continuous variables.

## Results

Table 1 illustrates the characteristics of the 26 patients who received the ICC since we first started using it in March 2016. Majority of the patients were ASA 2 or higher, and had 3 or more comorbidities. Most of our patients underwent major resections for tumours and lobectomy was most commonly performed. Mediastinal lymph node dissection was routinely performed in resections of primary lung malignancies. The total duration of surgery was 185.4 ± 81.4 min. A single chest drain was inserted in 25 patients (96.2%) and only one patient received 2 chest drains due to bilateral pleural effusions. None of our patients required extension of the UVATS incision to remove the resected specimen.

**Table 1:** Characteristics of patients who underwent UVATS and received the ICC

Variable	Value (n = 26)
Age (median and range)	61 (22–86)
Gender (no. of patients)	
Male	12 (46.2%)
Female	14 (53.8%)
Smoking history (no. of smokers)	19 (73.1%)
ASA^a^ score (no. of patients)	
1	2 (7.7%)
2	21 (80.8%)
3	3 (11.5%)
≥ 4	0 (0.0%)
Number of comorbidities (no. of patients)	
0	6 (23.1%)
1	5 (19.2%)
2	3 (11.6%)
≥ 3	12 (46.1%)
Preoperative diagnosis	
Primary lung cancer	19 (73.1%)
Secondary metastases	4 (15.4%)
Tuberculosis	1 (3.8%)
Cystic bronchiectasis	1 (3.8%)
Interstitial lung disease	1 (3.8%)
Surgical procedure	
Wedge resection	4 (15.4%)
Segmentectomy	6 (23.0%)
Lobectomy	14 (53.9%)
Metastasectomy	2 (7.7%)

^a^ASA = American Society of Anaesthesiologists

**Fig. 1:**
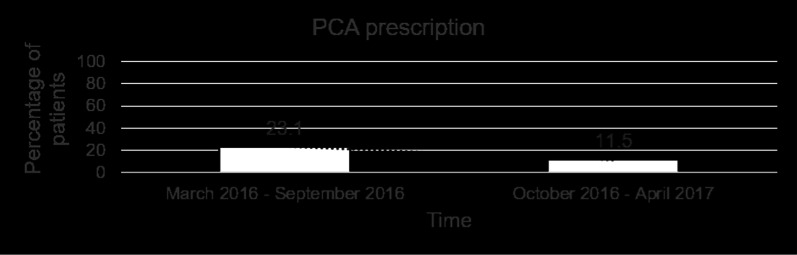
Percentage of patients who were prescribed morphine patient-controlled analgesia by the acute pain team

**Fig. 2:**
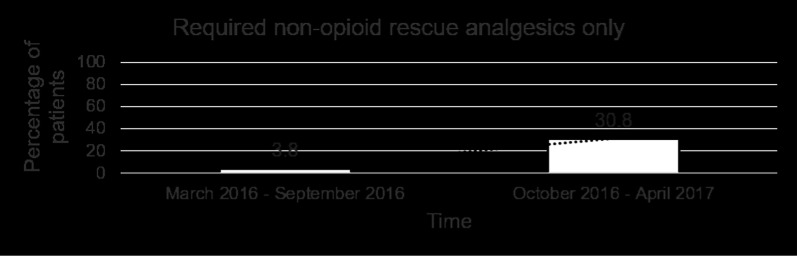
Percentage of patients who required non-opioid rescue analgesics only

**Fig. 3:**
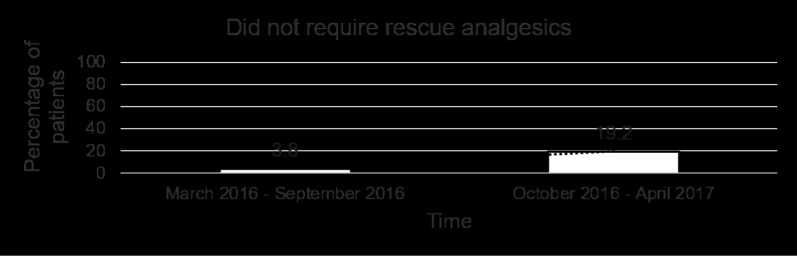
Percentage of patients who did not require rescue analgesics

Table 2 shows the mean pain scores over the first 5 postoperative days. In addition to receiving the standardised analgesic regimen (single-shot intercostal block and regular paracetamol) and the ICC, a total of 9 patients (34.6%) required PCA and 16 patients (61.6%) required a combination of rescue analgesics. 1 patient (3.8%) only received the single-shot intercostal block and ICC, and did not require regular paracetamol or any other rescue analgesics. Of those who were prescribed PCA, 7 of them used boluses amounting up to 5 mg only and therefore PCA was discontinued on POD 1. Of the remaining 2 patients who had PCA discontinued on POD 2, one had pre-existing rheumatoid arthritis. Amongst the 16 patients who required rescue analgesics, 7 of them required opioids as detailed in Table 3. The remaining 9 used combinations of etoricoxib and gabapentin. Over the time course of the study, the trend in PCA prescription progressively decreased (Fig. 1). This was accompanied by an increase in the percentage of patients who only required non-opioid rescue analgesics (Fig. 2), as well as an increase in the percentage of patients who did not require any rescue analgesics (Fig. 3). The ICC was left in-situ for a mean duration of 3.69 ± 0.47 days. 8 patients (30.8%) were pain-free by POD 3 and had their ICC removed before the pump was depleted. There was 1 incident (3.8%) of peri-catheter leakage which was rectified by placing Dermabond® (Ethicon) around the catheter insertion site and securing the catheter to the skin with Tegaderm™ to prevent accidental dislodgement. None of the patients experienced adverse effects related to ropivacaine toxicity (perioral numbness, hallucinations, hypotension, arrhythmias).

**Table 2:** Postoperative pain scores using the numeric rating scale (0–10)

Postoperative day	Pain score (mean and range)
1	0.31 (0–2)
2	0.31 (0–2)
3	0.23 (0–2)
4	0.12 (0–2)
5	0.00

**Table 3:** Number of patients who required opioid rescue analgesics

Analgesic combination	No. of patients
Paracetamol and tramadol	5
Paracetamol and morphine	1
Paracetamol and oxycodone	1

Secondary outcomes are presented in Table 4. 2 patients (7.7%) had empyema, of which one had a persistent air leak and therefore had a chest drain in situ for 25 days. This particular patient was discharged after 31 days of hospitalisation. None of the patients had atelectasis or pneumonia. There were also no non-pulmonary complications like urinary retention, deep venous thrombosis, surgical site infection or mortality.

**Table 4:** Secondary outcomes

Variable (median and range)	Value
Chest tube duration, days	3 (2–25)
Days to ambulation	1 (1–2)
Postoperative length of stay, days	3 (2–31)

## Discussion

With the introduction of muscle-sparing and nerve-sparing incisions in thoracic surgery, pain due to rib or intercostal nerve trauma have been shown to be minimized [17–19]. However, despite the usage of such techniques, significant acute postoperative pain has still been reported after UVATS [21, 22] and this is thought to be due to the irritation of the pleura or neurovascular bundles by chest tubes [23–25]. Pain management in thoracic surgery should be multimodal, with an aim to avoid or minimize the use of opioids [26]. Opioids are associated with adverse effects like nausea, drowsiness and respiratory depression which may delay postoperative recovery [24]. In the era of an increasing emphasis on Enhanced Recovery After Surgery (ERAS®), there are clear guidelines supporting the utility of regional analgesia to avoid the use of opioids [16].

Till date, there has been no consensus on the gold standard for regional analgesia after video-assisted thoracoscopic surgery (VATS), much less UVATS [27]. Prospective, randomized studies comparing TEA and PVB in VATS cases have demonstrated conflicting results on pain scores and opioid consumption, but consistently showed that TEA was associated with a higher frequency of hypotension and urinary retention [2, 4, 5]. This has resulted in PVB becoming more appealing than TEA, but studies have revealed that a continuous infusion technique rather than a single-shot blockade resulted in the decreased need for opioids and more optimal pain control [28, 29]. Being a form of regional analgesia that can provide a continuous infusion, the ICC became the subject of interest in a few observational studies which supported its utility in reducing pain and opioid usage after VATS [13, 30–32]. However, many of these studies have had mixed populations of single-port and multi-port VATS, thereby introducing heterogeneity in the available body of evidence. To the best of our knowledge, there was only one prior study focused on incorporating an ICC in UVATS exclusively, and the evidence of its utility in this domain remains sparse [33]. Aside from its relative novelty, our study is also distinctive in its inclusion of all consecutive patients who received the ICC since we first started using it in UVATS.

In our experience, the placement of an ICC provides adequate pain control after UVATS. In our earlier case series on muscle-sparing UVATS [34], we obtained mean pain scores of 0.2–0.4 on POD 1–5 before reaching 0.0 at POD 8. The inclusion of the ICC has improved our pain management, as evidenced by lower mean pain scores and earlier achievement of 0.0 on POD 5 in the present study. Our results also suggest that the ICC may assist with reduction in opioid usage. For most of our patients, a standardised regimen of an intercostal block and paracetamol, coupled with non-opioid rescue analgesics like etoricoxib and gabapentin, were adequate for pain management. These non-opioids were preferred due to their efficacy in controlling postoperative pain [35, 36]. In the small proportion of patients who required opioids, it is noteworthy that a weak-acting opioid like tramadol was sufficient. The frequency of PCA prescription also decreased over time when the acute pain team in our institution noted that minimal morphine boluses were required.

The ICC has multiple benefits in UVATS aside from pain control. Firstly, the catheter can be placed easily under direct vision, with confirmation of its placement by visualisation of an intercostal bulge after bolus administration of the analgesic. This is in contrast to TEA catheters which are inserted blindly and hence prone to mispositioning. The dosage of TEA also has to be titrated regularly which can be labour intensive. This is avoided with the ICC as the fixed pump volume and flow-limiting valve ensure an independent and constant flow rate. In addition, the ICC can be utilised when TEA is contraindicated, such as previous spine surgery or morbid obesity. Moreover, the ICC can deliver a continuous infusion beyond POD 1, thereby avoiding the short analgesic duration associated with single shot PVB. The ICC also enables early mobilisation since it is not associated with loss of motor function, as is the case with TEA. De Cosmo et al. [37] used an ICC-infusion regime similar to ours and reported a mean time to walking of 31.5 h, which is comparable to our findings. The portability of the ICC has also been demonstrated by Gebhardt et al. [38], who discharged 2 patients while the ICC was still in-situ. In our institution, we chose to err on the side of caution by removing the ICC in our patients before discharge. The median postoperative length of stay in our cohort was 3 days, which also compared favourably with the results by Ried et al. [39].

As with all equipment, technical failure is a concern but evidence on continuous anaesthetic infusion techniques like the ICC have shown the rate to be very low at 1% [40]. Our team’s specialised thoracic nurse reviewed all patients who received the ICC daily and would troubleshoot with the vendor if required. A potential drawback of the ICC is the risk of systemic toxicity. Jung and colleagues [32] reported that 3.3% of their patients who received the ICC complained of dizziness and drowsiness, with 2 patients requiring early discontinuation. It is our belief that with successful placement of the ICC confirmed intraoperatively, the risk of the analgesic entering systemic circulation is low. We further mitigated this risk by choosing to use an ICC with a fixed flow rate and clamp, to eliminate the possibility of increasing the flow rate accidentally and allow for quick cessation of the analgesic flow in an event of an emergency. A 16% rate of post-thoracotomy wound infection with the ICC has also been reported [39] but we did not observe the same findings, possibly because of the smaller incision in UVATS. Wheatley III and colleagues [41] claimed that the only contraindication to ICC is anaesthetic allergy and this is strongly supported by our team.

Our preliminary observational study was understandably not without its limitations. It was retrospective and based on a small sample size. We also acknowledge that the lack of a control group meant that our results should be interpreted with care. However, the use of a standardised ICC placement method, as well as a standardised analgesic regimen may have strengthened the validity of our findings. Pain score assessment was performed by uniformly trained nurses and analgesic usage was documented in a standardised manner in the electronic medical records, which may have helped to minimize biases in our study. Our findings have suggested that the ICC is simple and safe to utilize, and pain relief post-UVATS is also adequate. In the future, it is warranted to conduct larger-scale prospective randomized studies to further support the role of the ICC as regional analgesia in UVATS.

## Conclusions

Our initial experience with the ICC demonstrated that it is feasible for incorporation into a multimodal analgesic strategy for UVATS. Its usage appears to be associated with minimal post-operative pain and reduced usage of opioids. It can therefore be considered as an adjunct to achieve early recovery after UVATS.

## Data Availability

The datasets used and/or analysed during the current study are available from the corresponding author on reasonable request.

## Abbreviations

ASA: American society of anaesthesiologists
ERAS: Enhanced recovery after surgery ®
HDU: High dependency unit
ICC: Intercostal catheter
PCA: Patient controlled analgesia
POD: Postoperative day
PVB: Paravertebral block
SPSS: Statistical package for the social sciences
TEA: Thoracic epidural analgesia
UVATS: Uniportal video-assisted thoracoscopic surgery
VATS: Video-assisted thoracoscopic surgery
